# SARS-CoV-2 Lineage Tracking, and Evolving Trends Seen during Three Consecutive Peaks of Infection in Delhi, India: a Clinico-Genomic Study

**DOI:** 10.1128/spectrum.02729-21

**Published:** 2022-03-21

**Authors:** Pramod Gautam, Diptanu Paul, Varun Suroliya, Rahul Garg, Reshu Agarwal, Santanu Das, Urvinder S. Kaur, Amit Pandey, Arjun Bhugra, Bansidhar Tarai, Chhagan Bihari, S. K. Sarin, Ekta Gupta

**Affiliations:** a Genome Sequencing Laboratory, Institute of Liver and Biliary Sciences, New Delhi, India; b Department of Clinical Virology, Institute of Liver and Biliary Sciences, New Delhi, India; c Department of Pathology, Institute of Liver and Biliary Sciences, New Delhi, India; d Max Super Speciality Hospital, Max Healthcare, New Delhi, India; e Department of Hepatology, Institute of Liver and Biliary Sciences, New Delhi, India; Johns Hopkins Hospital

**Keywords:** SARS-CoV-2 variants, COVID-19, pandemic, lineage tracking, Delhi, India, COVID-19 in Delhi, COVID-19 tracking, genome surveillance, SARS-CoV-2 lineages

## Abstract

Since its advent, the pandemic has caused havoc in multiple waves due partly to amplified transmissibility and immune escape to vaccines. Delhi, India also witnessed brutal multiple peaks causing exponential rise in cases. Here we had retrospectively investigated clade variation, emergence of new lineages and varied clinical characteristics during those three peaks in order to understand the trajectory of the ongoing pandemic. In this study, a total of 123,378 samples were collected for a time span of 14 months (1 June 2020 to 3 August 2021) encompassing three different peaks in Delhi. A subset of 747 samples was processed for sequencing. Complete clinical and demographic details of all the enrolled cases were also collected. We detected 26 lineages across three peaks nonuniformly from 612 quality passed samples. The first peak was driven by diverse early variants, while the second one by B.1.36 and B.1.617.2, unlike third peak caused entirely by B.1.617.2. A total of 18,316 mutations with median of 34 were reported. Majority of mutations were present in less than 1% of samples. Differences in clinical characteristics across three peaks was also reported. To be ahead of the frequently changing course of the ongoing pandemic, it is of utmost importance that novel lineages be tracked continuously. Prioritized sequencing of sudden local outburst and community hot spots must be done to swiftly detect a novel mutation/lineage of potential clinical importance.

**IMPORTANCE** Genome surveillance of the Delhi data provides a more detailed picture of diverse circulating lineages. The added value that the current study provides by clinical details of the patients is of importance. We looked at the shifting patterns of lineages, clinical characteristics and mutation types and mutation load during each successive infection surge in Delhi. The importance of widespread genomic surveillance cannot be stressed enough to timely detect new variants so that appropriate policies can be immediately implemented upon to help control the infection spread. The entire idea of genomic surveillance is to arm us with the clues as to how the novel mutations and/or variants can prove to be more transmissible and/or fatal. In India, the densely populated cities have an added concern of the huge burden that even the milder variants of the virus combined with co-morbidity can have on the community/primary health care centers.

## INTRODUCTION

Severe acute respiratory syndrome coronavirus-2 (SARS-CoV-2), a novel human *betacoronavirus*, was declared as a causative agent for global pandemic of the disease Coronavirus disease 2019 (COVID-19) on11^th^ March 2020, by World Health Organization (WHO) (1, 2). In India, the first case of SARS-CoV-2 was reported on 27^th^ January 2020 (3). Following this, the country witnessed two major waves of infection in August and September 2020 and April and May 2021 unlike National Capital Region, Delhi with three peaks of infection reported in May–July 2020, November-December 2020, and later on a massive and deadly one in April–June 2021 (4, 5). A sudden surge in cases of SARS-CoV-2 in a population rests upon several factors among which emergence of novel mutations in the viral genome leading to the appearance of possibly more virulent and fatal variants is a huge cause for concern in this ongoing pandemic (6). In India, as has been observed by many studies, the spread of COVID-19 infection is attributable to rising novel variants and their spread (7, 8). Studying SARS-CoV-2 genomic variants and their tracking with time might help us in understanding viral evolution, behavior, and infection trajectory. To put a break on the possible eventual waves of infection, continuous tracking on emerging mutations leading to novel viral lineages and their clinical impact is of paramount importance.

Hence, a comparison between three peaks of infection in Delhi capital region is the focus of the present study. Here we have described clade variation, emergence of new lineages and varied clinical characteristics to understand the trajectory of the ongoing pandemic.

## RESULTS

Among 612 samples, 560 fell into the defined time period of peak definition. A total of 98 (16%) samples were collected during first peak, 83 (13.5%) during second peak and 379 (61.9%) during the third peak (Table 1). The remaining 52 samples were collected either just before (pre-third peak) or after Peak 3 (post-third peak).

**TABLE 1:** Clinical and demographic details across three peaks

Parameter	Groups	1st peak (*N* = 98)	2nd peak (*N* = 83)	3rd peak (*N* = 379)	*P*-value^*a*^
Sex	Males	58 (59.2%)	48 (57.8%)	228 (60.2%)	0.92
	Females	40 (40.8%)	35 (42.2%)	151 (39.8%)	
Age	Age group I (0–10 yr)	31 (31.6%)	13 (15.6%)	15 (3.9%)	0.03*
	Age group II (11–25 yr)	25 (25.5%)	38 (45.7%)	120 (31.6%)	5.15 × 10^−6^***
	Age group III (26–50 yr)	25 (25.5%)	20 (24.1%)	184 (48.8%)	0.89
	Age group IV (≥51 yr)	17 (17.3%)	12 (14.4%)	60 (15.8%)	0.56
Clinical symptoms	Symptomatic	48 (49%)	48 (57.8%)	245 (64.6%)	0.014**
	Asymptomatic	50 (51%)	35 (42.2%)	134 (35.4%)	
Severe acute respiratory illness	Yes	5 (5.1%)	7 (8.4%)	54 (14.2%)	0.025*
	No	93 (94.9%)	76 (91.6%)	325 (85.8%)	
Influenza-like illness	Yes	43 (43.9%)	41 (49.4%)	191 (50.3%)	0.51
	No	55 (56.1%)	42 (50.6%)	188 (49.6%)	
Outcome	Survived	95 (96.9%)	81 (97.6%)	341 (90%)	0.01**
	Deceased	3 (3.1%)	2 (2.4%)	38 (10%)	
Ct value (S gene)	Low	85 (86.7%)	61 (73.5%)	141 (37.2%)	1.6 × 10^−14^***
	Medium	13 (13.2%)	19 (22.9%)	214 (56.4%)	0.27
	High	0 (0%)	3 (3.6%)	24 (6.3%)	0.69

a ***, ≤ 0.001; **, ≤ 0.01; *, ≤ 0.05.

Whenever peak-wise comparison was made, these 560 samples only were taken into consideration. For rest of the analyses, total 612 sequences samples were considered.

### Demographic and clinical data.

Complete demographic and clinical details along with comparison across different peaks are as follows:

**(i) Overall clinical and demographic details.** Among 612 cases, 60.4% (*n* = 370) were males and 39.54% (*n* = 242) were females. The age-wise distribution was as follows: 67 cases (0–10 yr), 210 cases (11–25 yr), 237 cases (26–50 years) and 98 cases (>50 yr). Majority of the enrolled cases (60.4%, *n* = 370) were symptomatic. Among these, severe acute respiratory illness (SARI) was recorded only in 19% (*n* = 70) and remaining cases presented with influenza-like illness (ILI). Fatal outcome was reported in 7.5% (*n* = 46) cases.

**(ii) Peak-wise comparison of clinical and demographic details.** Males were more affected than females, but no significant difference was found across peaks. For age group I (0–10 yr), a significant difference was observed between three peaks (*P* = 0.03). Similarly, more cases belonging to age group II (11–25 years) were reported in third peak compared with first peak (*P* < 0.001) and second peak (*P* = 0.001) (Fig. S1 in the supplemental material). Fraction of symptomatic cases gradually rose from 49% in first peak to 65% in third peak with statistically significant difference (*P* = 0.01). Maximum SARI cases were recorded during the third peak (*P* = 0.02). No significant difference in proportion of ILI cases was seen across the peaks (Table 1). The mortality rate was considerably higher (10%) during the third peak than the first (3.1%), and the second peak (2.4%). The specimen with low Ct values was found to be significantly higher in third peak possibly indicating higher viral load during infection. The median Ct value during the first two peaks were 15.3 and 17.7, respectively, but during third peak, it was 21.6. (Fig. S2).

### Diversity and distribution of SARS-CoV-2 lineages.

A total of 26 different lineages were identified from 612 sequences following PANGO (Phylogenetic Assignment of Named Global Outbreak) nomenclature (Table S4 in the supplemental material).

**(i) Trends of various circulating lineages across peaks.** The most frequently detected lineages in our data set were B.1.617.2 including its sub-lineages (*n* = 369, 60.3%) and B.1.36 (*n* = 90, 14.7%). However, some of the lineages were observed to emerge and wane with each emerging peak.

**FIG 1:**
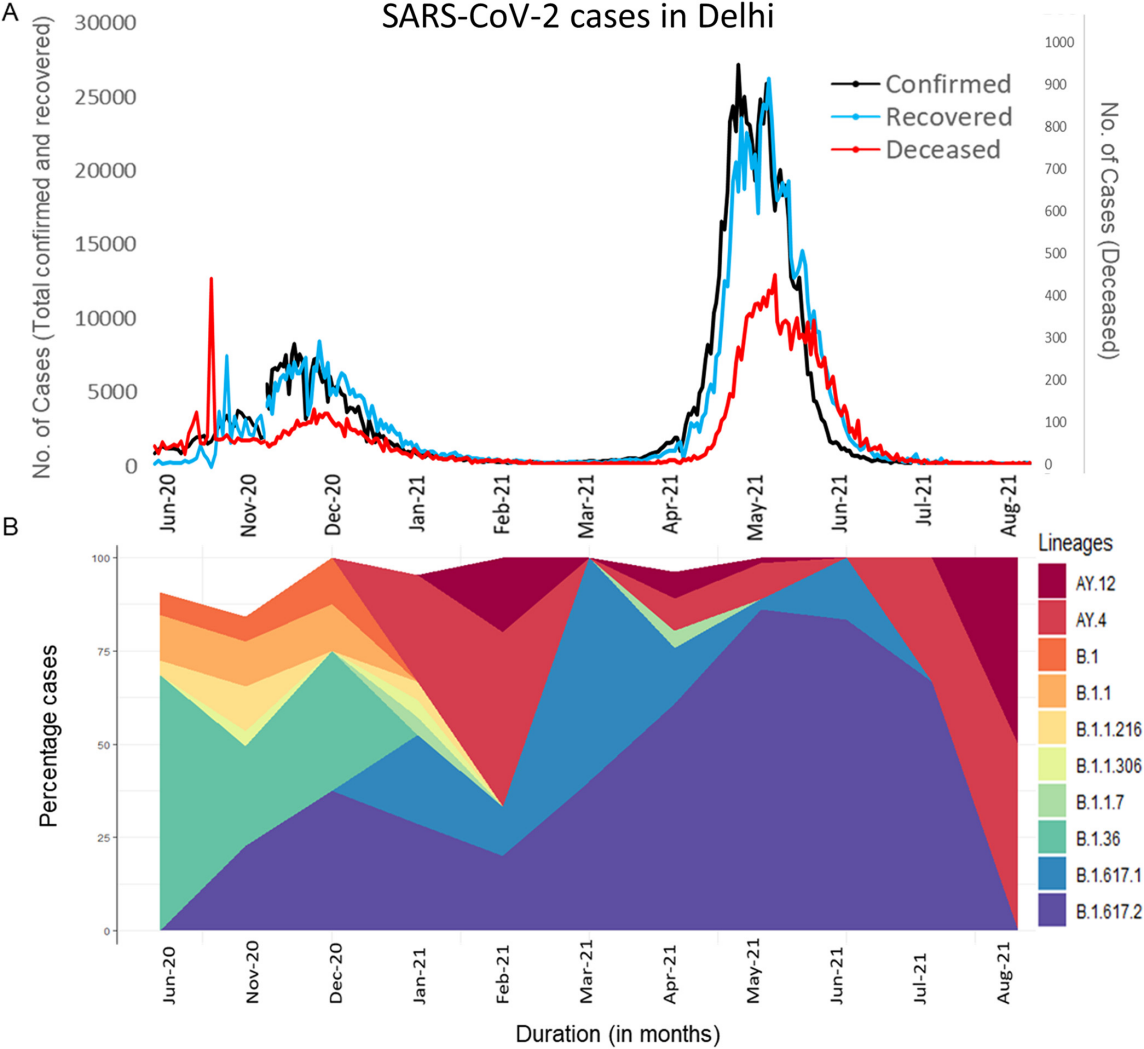
Trend of SARS-CoV-2 lineages in Delhi. A: Daily new cases, recovered cases and deaths reported in Delhi (https://github.com/covid19india/api.git). B: Trend of the 10 most detected lineages in the present study.

During the first peak, a total of 11 different lineages were identified. The most common lineage was B.1.36 (*n* = 67, 68.40%) followed by B.1.1 (*n* = 12, 12.25%). During the second peak, 13 different lineages were identified, among which B.1.36 was the predominant (*n* = 23, 27.71%) followed by B.1.617.2 (*n* = 20, 24.09%). Four lineages (B.1, B.1.1, B.1.36, and B.1.1.216) continued to be detected during this peak also. There is a substantial rise of B.1.617.1 immediately after the end of second peak along with B.1.617.2. However, during the third peak the overall diversity of circulating lineages decreased to only 8. The B.1.617.2 variant overpowered other circulating lineages and became the most predominant lineage (*n* = 311, 82.05%), during the third peak (Fig. 1). The other predominant lineages were – B.1.617.1 (*n* = 58, 13.46%), B.1.1.7 (*n* = 15,3.49%) and B.1.617.3 (*n* = 5, 1.16%). Lineages detected during 1st peak (B.1, B.1.1, and B.1.1.216) were not frequent during third peak. B.1.306 and B.1.456 were shared between second and third peak. B.1.216 was detected in all three peaks. However, these three lineages were found in only one samples each, during third peak.

Post second peak, three novel sub-lineages of B.1.617.2, (AY.6, AY.12, and AY.4) started emerging. During third peak, these sub-lineages accounted for 16% of the total lineages in circulation (AY.4, *n* = 32, 8.7%), AY.12 (*n* = 22, 6%), AY.6 (*n* =6, 1.6%). All lineages detected following third peak belonged to either B.1.617.2 or its sub-lineages.

**FIG 2:**
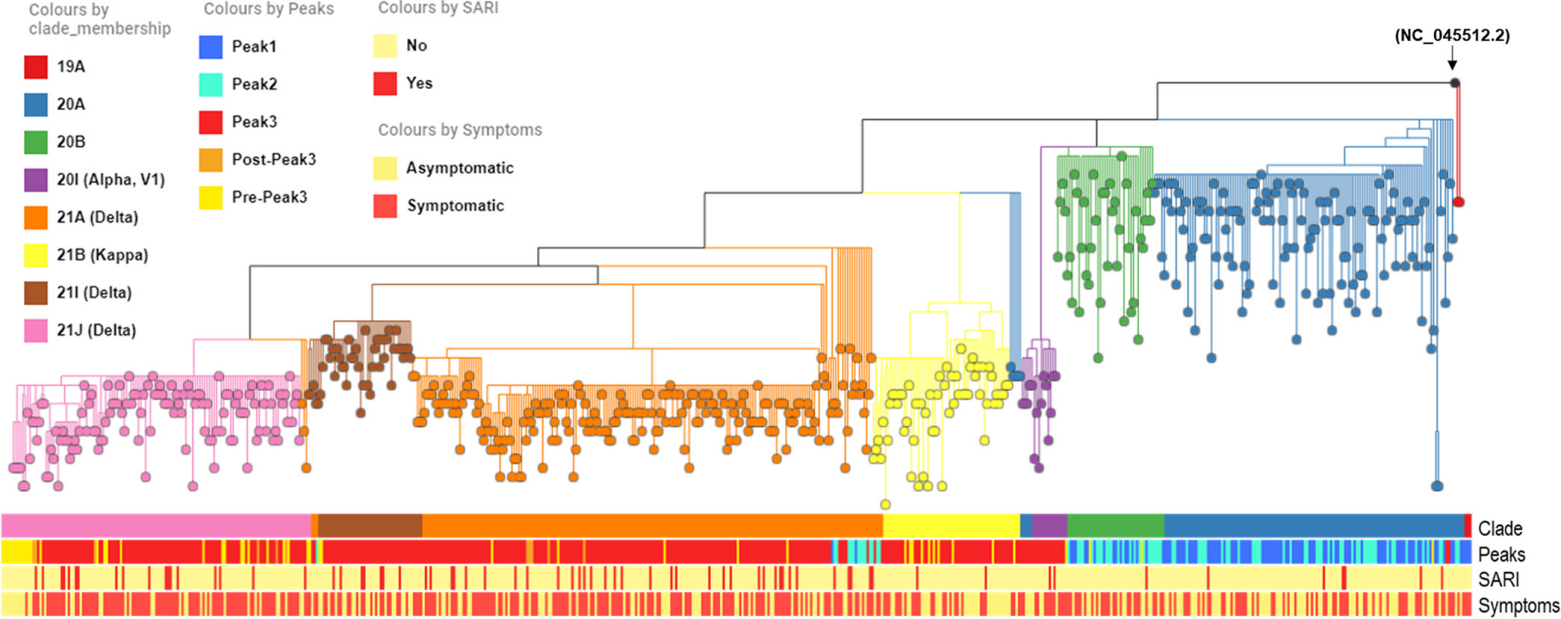
Phylogenetic tree of detected lineages. The tree is rooted along Wuhan-Hu-1 (NCBI Reference Sequence: NC_045512.2) as reference (denoted by black node). The four horizontal color panels represent the corresponding clade membership, peaks period, SARI and overall symptomatic relationship respectively.

**(ii) Evolutionary relationship among detected lineages.** The phylogenetic relationship of all the lineages detected in our data set shows eight major clade members as per Nextstrain clade assignment (9). Three different Delta variant lineages (21A, 21I and 21J) were present along with 20A, 20B and 20I (Alpha, V1) and 21B (Kappa) with 19A occurring rarely (Fig. 2).

### Mutational spectra of SARS-CoV-2 during peaks.

**FIG 3:**
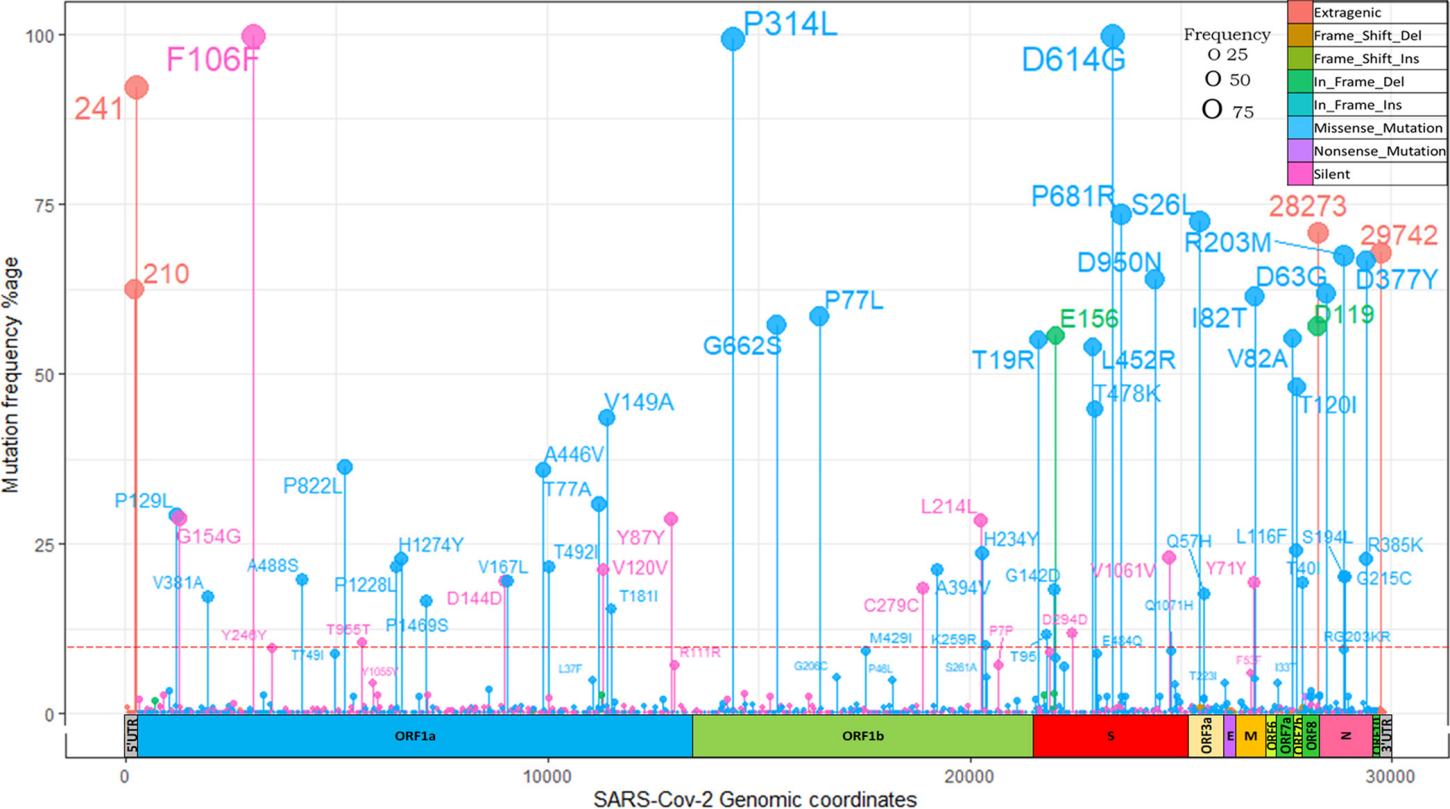
Mutational landscape of 612 samples. *x* axis shows the genomic coordinates of the SARS-Cov-2 virus. *y* axis depicts the frequency of detected mutation, color coded by their types. The horizontal dotted red line denotes the 10% frequency cutoff.

Along with the mutational burden we also investigated their genomic location, frequencies, and eventual impact on amino acid sequences (Fig. 3). A total of 18,316 mutations with median of 34 mutations/sample were recorded (Table S5 in the supplemental material). A total of 1,803 mutations were present nonredundantly. Out of these, 156 mutations (9%) were present in at least 1% of samples and remaining 91% were rarely detected. Only 21 mutations were found to be present in at least half of the samples. Most commonly observed category was nonsynonymous mutations.

**FIG 4:**
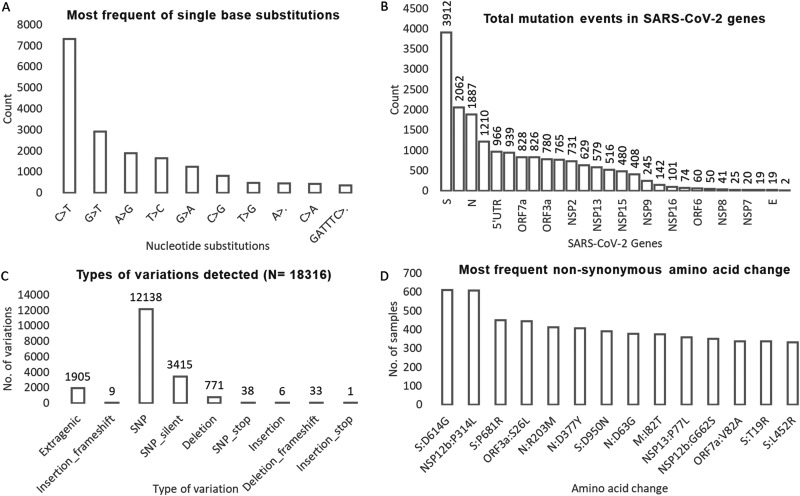
The mutations detected and their distribution with respect to their type, genomic location, and frequency. A: The plot shows the most frequent single base substitutions present in our data. B: The total mutation events in different genic and nongenic regions of SARS-CoV-2. C: Type of mutations observed. D: Most frequent nonsynonymous amino acid changes observed.

The most common type of base substitution observed was C > T which accounted for 40% of all the changes detected (Fig. S3 in the supplemental material). Among the 10 most frequent nucleotide mutations, the only genic deletion observed was of 6 bp region (GATTTC) in ORF8 gene (Fig. 4A). The gene which was found to harbor the greatest number of mutations was S gene (21% of all mutations), followed by NSP3 gene (11%) and N gene (10%) respectively (Fig. 4B and Fig. S4). Two-thirds (*n* = 12,138) of all the detected mutations were nonsynonymous SNPs followed by synonymous SNPs (*n* = 3,415) (Fig. 4C). The most common mutations that we observed in our data set were S: D614G, NSP3:F106F, NSP12b:P314L, and 5'UTR: 241 (all present in more than 90% of the sequences across all peaks) (Figs. 3 and 4D).

**FIG 5:**
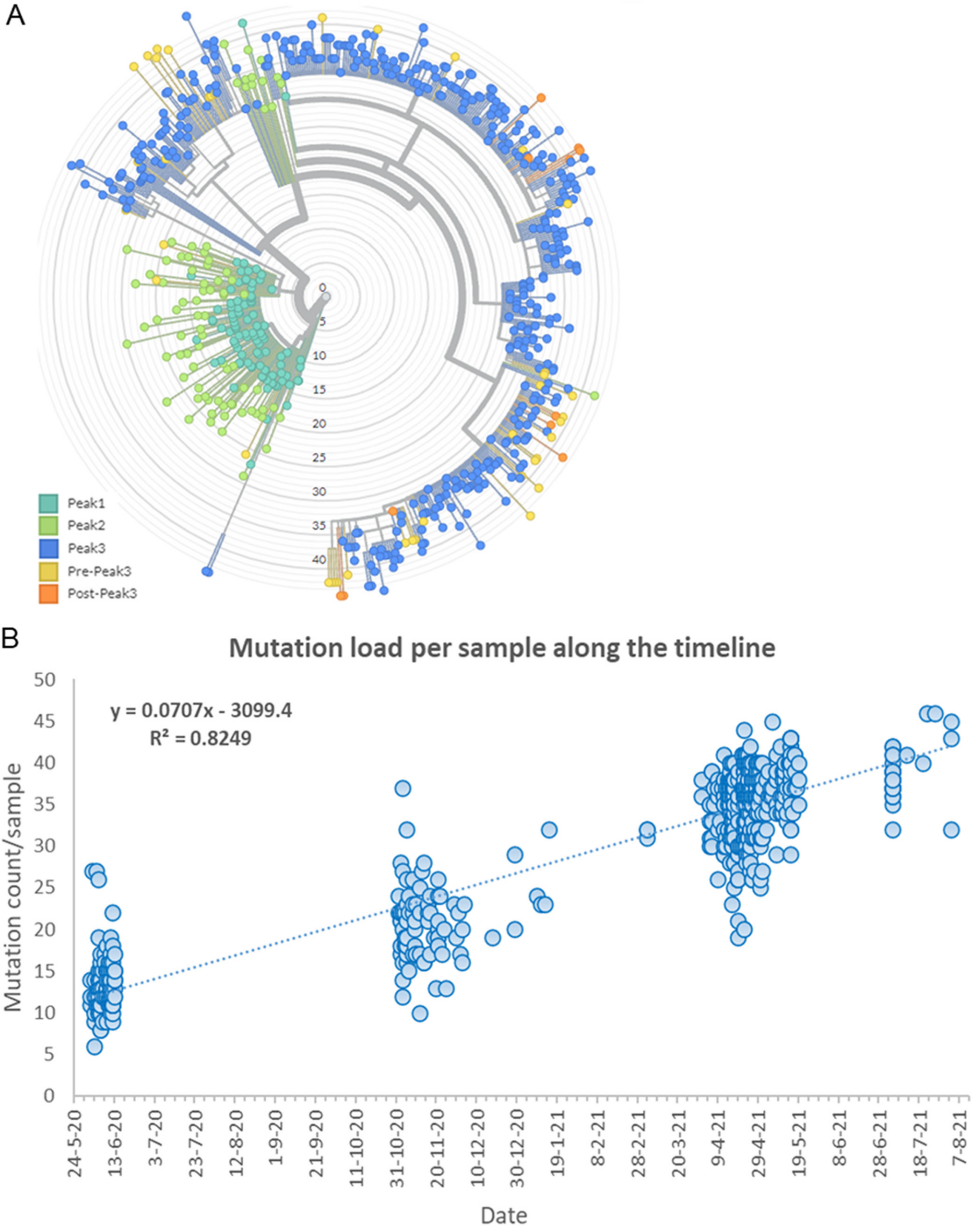
The phylogenetic relationship and mutation burden across different peaks. A: Lineages and their relationship with respect to each peak. The nodes are colored according to different peaks. The radial axis shows the total number of mutations in a lineage. B: Mutational burden per sample in different peaks. Strong correlation between mutational load and each consecutive peak is demonstrated (*r*^2^ = 0.82).

In terms of mutation load, samples from first peak contained expectedly lesser number of total mutations compared to subsequent peaks. The median number of total mutations during first peak was found to be only 13 which further increased to 20 mutations/sample during second peak and to much higher number of 36 mutations/sample (2.8 times compared to first peak) during third peak (Fig. 5).

**FIG 6:**
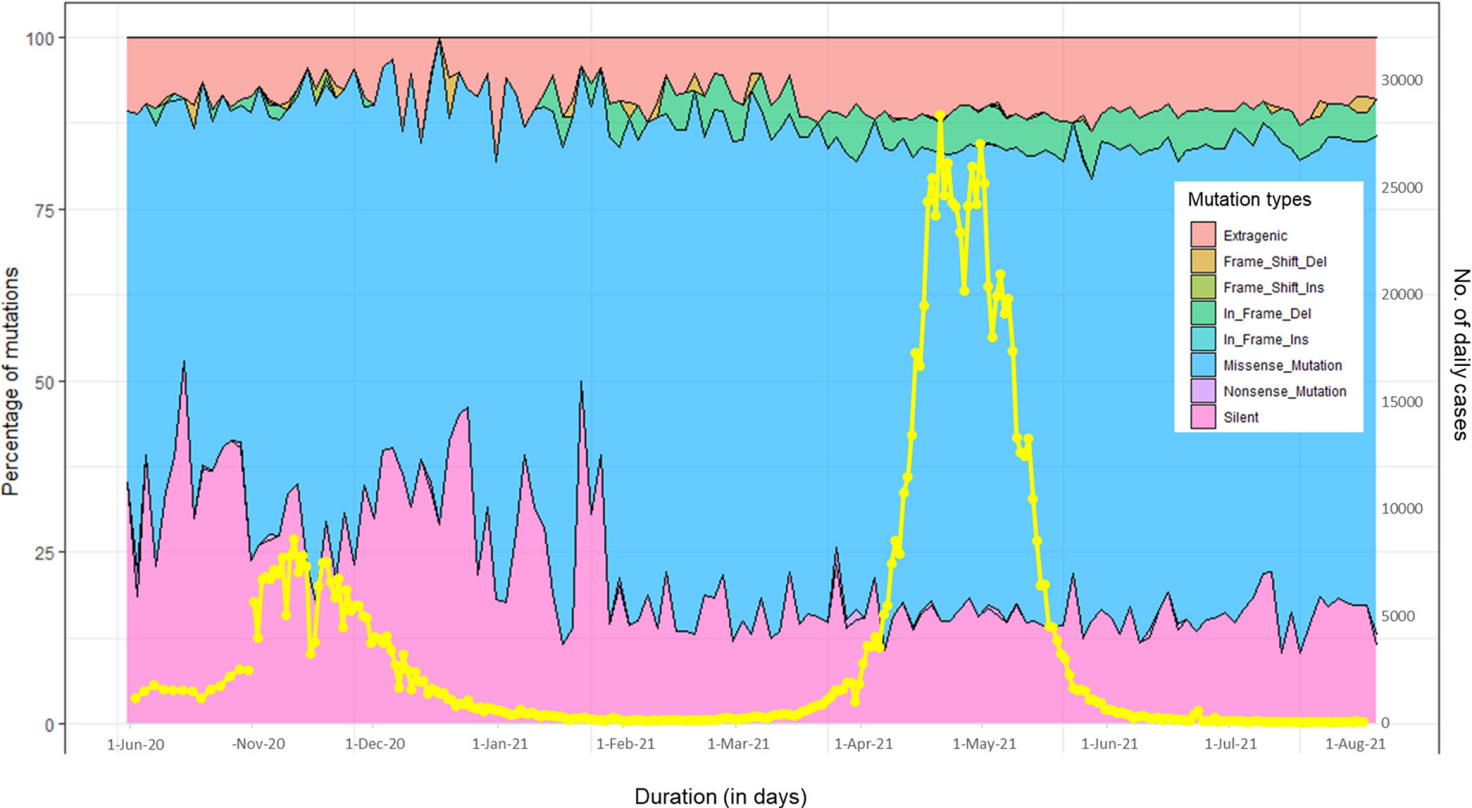
Drift in the number and types of mutations detected daily during the three peaks in Delhi. The stacked area plot shows the types of mutations detected in sequenced samples per day and line plot (yellow) depicts the overall case burden in Delhi. *x* axis depicts the time duration (in days). *y* axis (left-hand) depicts fraction of mutation types. *y* axis (right-hand) depicts number of daily infected cases in Delhi (https://github.com/covid19india/api.git).

As depicted in Fig. 6, apart from substantial increase in mutational load, proportional change in mutation type was also observed with each peak.The fraction of synonymous amino acid mutations decreased with each advancing peak (36% of all mutations in first peak to 16% during third peak). However, fraction of nonsynonymous SNPs/missense mutations gradually increased with time (53% during first peak to 68% during third peak). The In-frame deletion were present in less than 0.5% of cases daily during first and second peaks and jumped to approximately 5% cases daily (10 times increase) in third peak (Fig. 6).

### Comparison between common circulating lineages.

Two of the most frequently detected lineages(B.1.36 and B.1.617.2) in the present study were compared in terms of clinical characteristics and associated mutations.

**(i) Comparison of clinical characteristics.** For age group I (0-10 years), significantly more cases (24.4%) were affected by B.1.36 compared to B.1.617.2 (5.5%) (Table 2). In contrast, no significant difference was found for cases belonging to all the other age groups across the two lineages. A greater proportion of cases were symptomatic when infected with B.1.617.2 compared to B.1.36. Similarly, ILI and SARI was also significantly associated with B.1.617.2. A higher mortality rate was reported among B.1.617.2 cases (*P* < 0.05).

**TABLE 2:** Comparison of demographic and clinical parameters between B.1.36 and B.1.617.2 lineages

Parameter	Groups	B.1.36 lineage	B.1.617.2 lineage	*P* value
Sex	Males	59 (65.6%)	176 (61.1%)	*P* = 0.52
	Females	31 (34.4%)	112 (38.8%)	
Age	Age group I (0–10 yr)	22 (24.4%)	16 (5.5%)	*P* = 0.02*
	Age group II (11–25 yr)	23 (25.5%)	104 (36.1%)	*P* = 0.17
	Age group III (26–50 yr)	32 (35.5%)	124 (43%)	*P* = 0.602
	Age group IV (≥51 yr)	13 (14.4%)	44 (15.3%)	*P* = 0.72
Clinical symptoms	Symptomatic	31 (34.4%)	188 (65.3%)	*P* = 1 × 10^−4^***
	Asymptomatic	59 (65.6%)	100 (34.7%)	
Severe acute respiratory illness	Yes	3 (3.3%)	56 (19.4%)	*P* = 4 × 10^−4^***
	No	87 (96.7%)	232 (80.6%)	
Influenza-like illness	Yes	28 (31.1%)	132 (45.8%)	*P* = 0.01**
	No	62 (68.9%)	156 (54.1%)	
Outcome	Survived	89 (98.9%)	250 (86.8%)	*P* = 2 × 10^−3^**
	Deceased	1 (1.1%)	38 (13.2%)	

**FIG 7:**
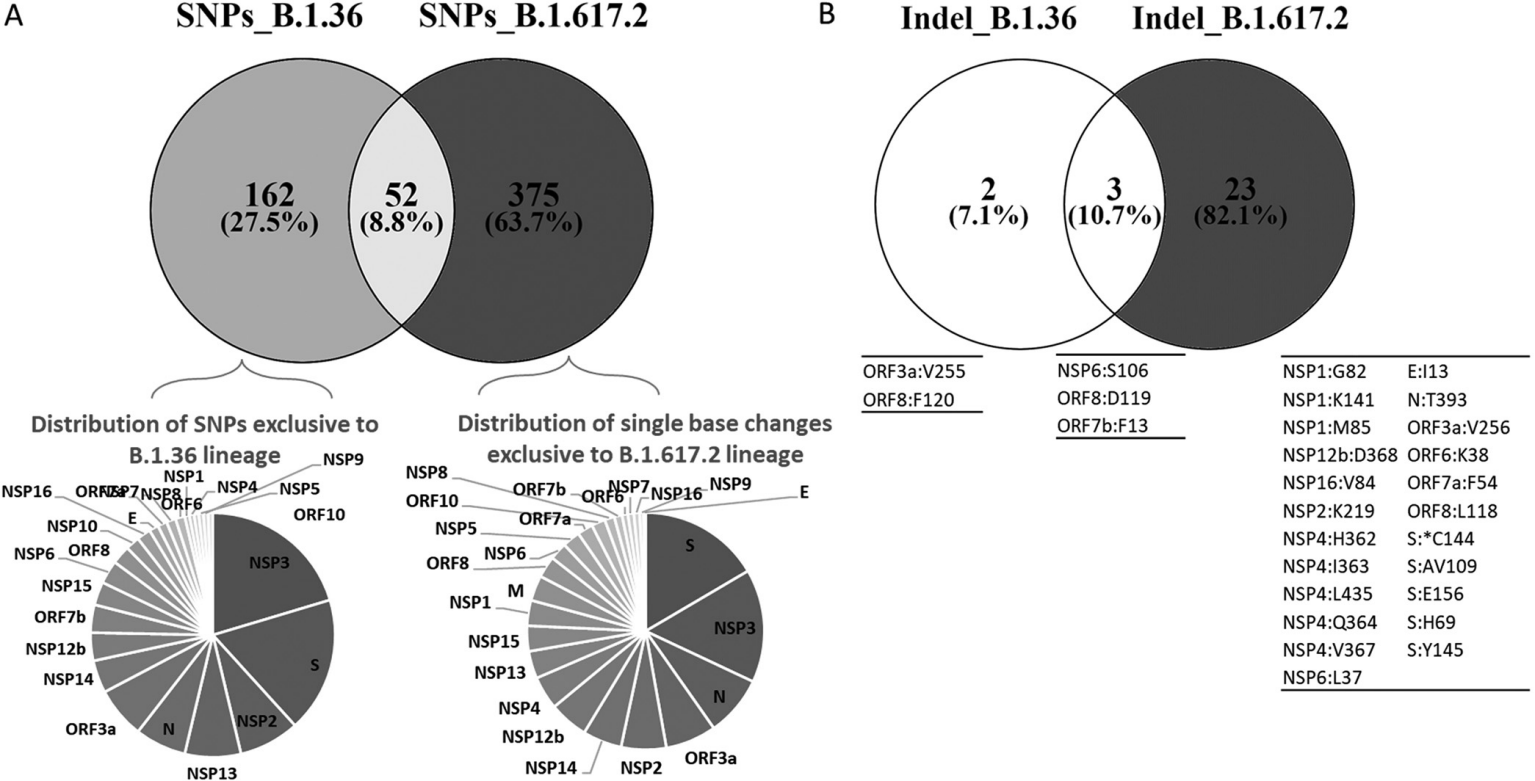
Comparison of mutations between B.1.36 and B.1.617.2 lineages. A: Nonsynonymous SNPs present in both lineages. B: The comparison of indel events present in both lineages.

**(ii) Comparison of mutation profile.** The number of nonsynonymous amino acid changes were found to be more in B.1.617.2 (63.7% vs 27%). Indel events were majorly present in B.1.617.2 (82.1%) compared to B.1.36 (7.1%) (Fig. 7).

**FIG 8:**
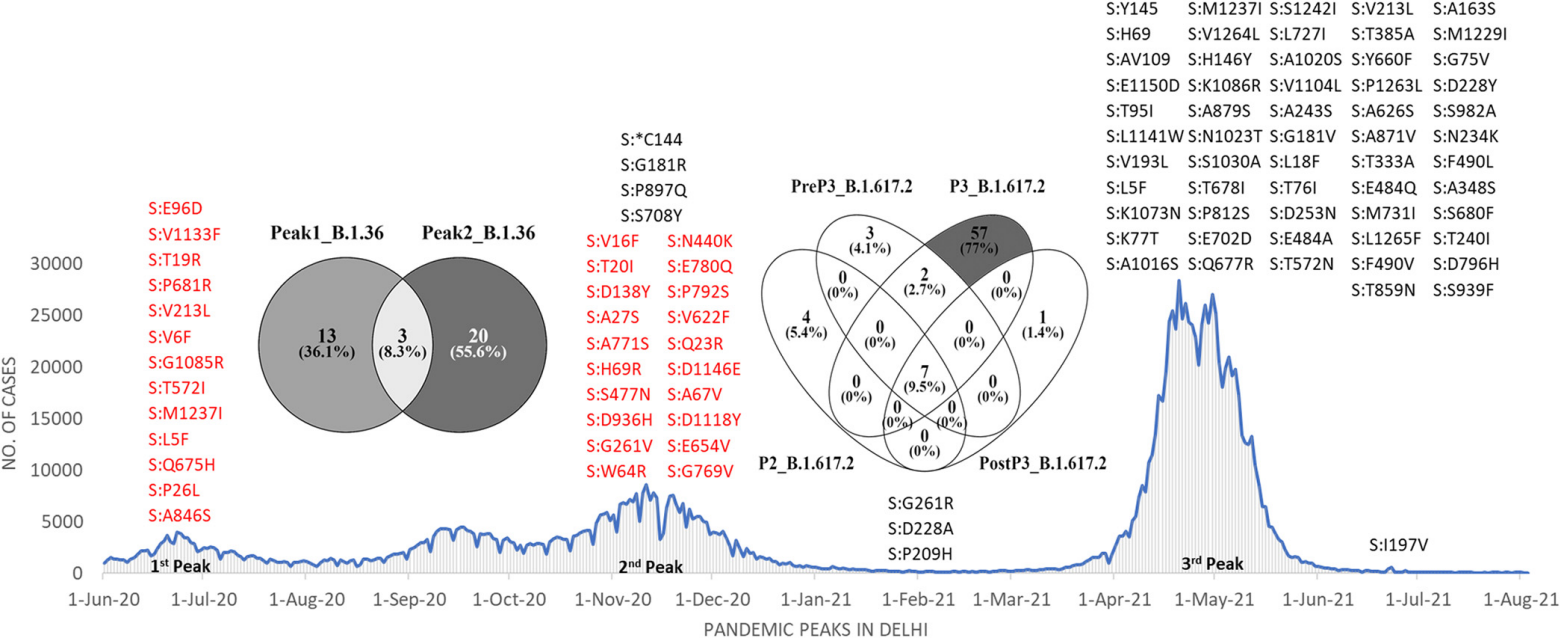
Exclusive S gene mutations detected in both lineages across different peaks. The mutations in B.1.36 and B.1.617.2 are coded in red and black, respectively.

S gene was found to be more prone to nonsynonymous changes in both lineages but with certain differences. Out of all S gene mutations detected in B.1.36, only 36.1% (*n* = 13) was present exclusively during the first peak when this lineage was a predominant one. Interestingly B.1.36 acquired novel set of mutations (55.6%) exclusive to the second peak. Similarly in B.1.617.2, only 5.4% (*n* = 4) and 4.1% (*n* = 3) were exclusively present during second and pre third peak period respectively. During third peak B.1.617.2 acquired 77% (*n* = 57) novel mutations. Only one novel mutation was acquired following 30 days after third peak had subsided (Fig. 8).

## DISCUSSION

This study combines the genomic information about the circulating lineages, associated variations along with varied clinical details to understand the evolution dynamics of SARS-CoV-2. The present study is first of its kind where we have tracked the SARS-CoV-2 mutation repertoire for a time span of 14 months encompassing all the three peaks of infection in Delhi.

In this study overall, 60.46% of the cases was male, which is expected as they were more prone to exposure due to their livelihood. The median age was approximately 17 years during first two peaks which jumped to 31 years during the third peak. When looked within each age bracket, we found no significant difference between age groups III (26–50 years) and IV (≥51 yr) but during third peak more patients belonging to age groups I (0–10 years) and II (11–25 years) were observed. This can be attributed to the fact that vaccination was not recommendatory for these subsets of susceptible population in India at the time when they got infected. This is in concordance with other studies where younger population was more affected during later peaks (10, 11). During third peak, more symptomatic cases and higher mortality rate was observed. The third peak as we have seen was dominated by the Delta lineage compared to the other lineage (B.1.36) of earlier peaks. These findings were corroborated by other multicentric studies (11, 12).

The Ct value is inversely proportional to the sample’s viral load, and we noted some significant difference in Ct value between the three peaks. We looked at the difference in Ct value in two ways, firstly whether the overall median Ct across peaks varied or not. We found that the median Ct value during the first two peaks were 15.3 and 17.7, respectively, but during third peak, it jumped to 21.6. It could be because of number of reasons like people not getting themselves tested promptly, or the saturation of the testing capacity of the labs during extremely high case load of third peak which could also have delayed the testing for a fraction of infected patients. Secondly, we grouped the samples in low, medium, and high Ct value groups and looked at the difference across peaks. Here we noted that for medium and high Ct values groups we did not find any significant difference between three peaks. The absence of low Ct value samples during the first peak could be because of prompt testing. In the low Ct group however, we did observe significant difference between the third peak and previous peaks (Fig. S2 in the supplemental material).

In our study, a total of 26 identified lineages dynamically varied over time. This temporal shifting in lineages is well documented across the world (13, 14). During the first peak, we observed multiple different lineages contributing to an abrupt rise in daily cases. Initially, the overall number of lineages was very high which further decreased with time indicating certain lineages became predominant and thus majorly contributed toward case rise. For example, B.1.36, which was a major player during the first peak (68.40%), declined to 27.71% during second peak and finally became undetected. Lineage B.1.617.2 emerged during second peak and was solely responsible for a deadly third peak in Delhi. Soon after second peak B.1.617.2 started accumulating more mutations ultimately giving rise of diverse sub-lineages (AY.x) which are proving to be a potential threat. After the third peak, B.1.617.2 and its sub-lineages has overpowered all other lineages. At the time of writing, as many as 33 different sub-lineages of the Delta variant have been identified (15). In our study, after the second peak subsided, we have found sub-lineage AY.4 in 11.4% (*n* = 49), AY.12 in 6% (*n* = 26), and AY.6 in 1.4% (*n* = 6) of samples.

**FIG 9:**
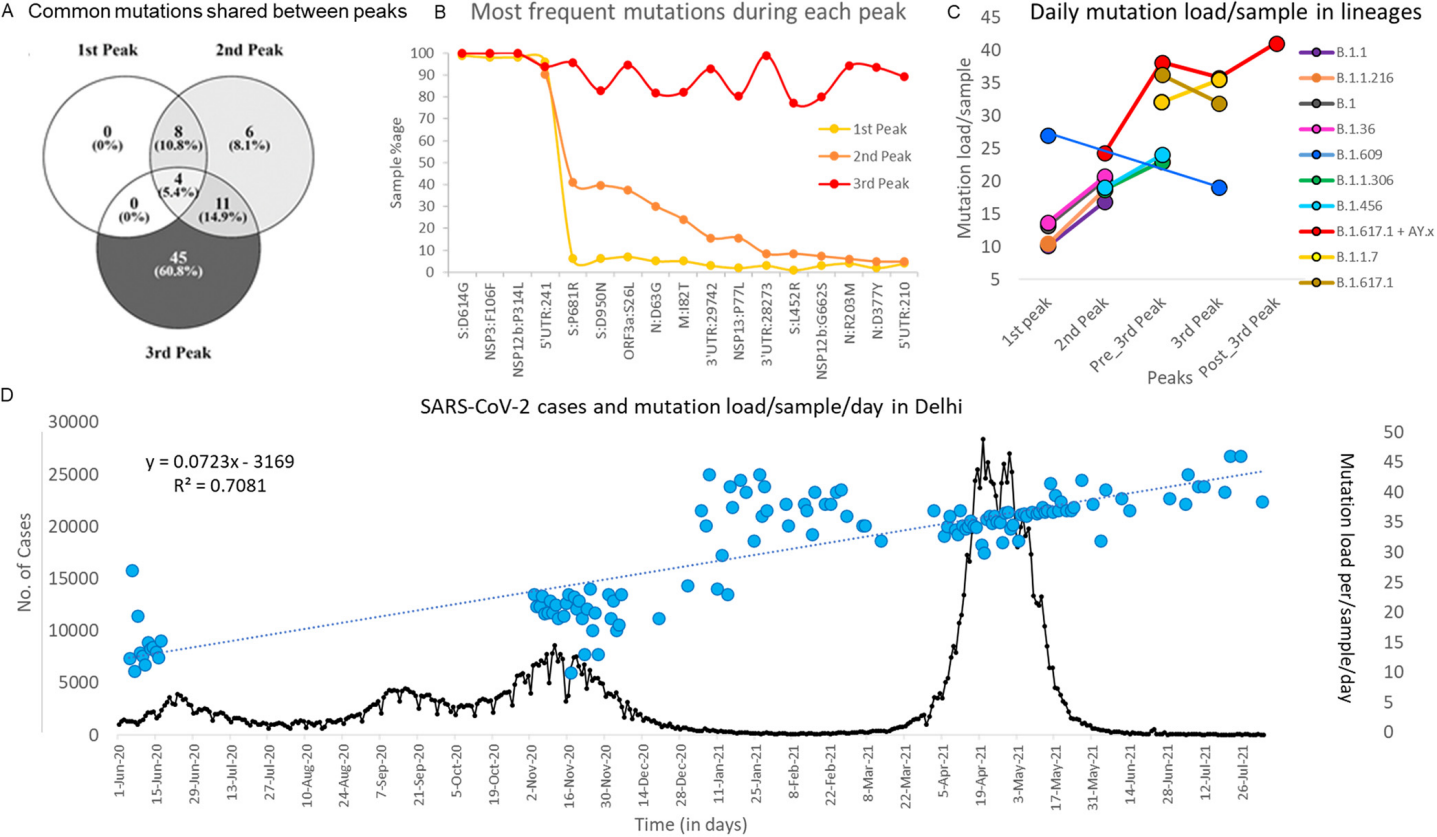
Common mutations across different peaks. A: The shared mutations between all three peaks. All types of mutations present in at least 10% of samples in a peak are considered. B: The topmost common mutations during each peak. The third peak was found to contain 17 very frequent mutations compared with 4 in first and second peak. C: Change in average mutation load/sample in lineages across peaks. Similar lineages in different peaks were found to have increased mutation load with time. D: The mutational burden on a per sample basis as observed during three different peaks. The linear trend line is given as dotted blue line. Mutation load/sample on each day is a blue solid blue circle (total days of collection = 133) and plot in black shows the daily infection cases in Delhi during the same time period (https://github.com/covid19india/api.git).

Because of concerns of increased transmissibility and/or mortality, it is imperative to be vigilant about novel mutations and their biological effects (13, 14, 16). In our study, mutation landscape of 612 samples showed that not only mutation types but also absolute number of mutation/sample changes with each peak. When we looked at the commonly occurring mutations (mutation circulating in more than 10% of the sequenced data for a given peak) irrespective of their type and lineages, it was found that 81% of these frequent mutations were in circulation during third peak. Out of which, almost 61% were exclusively present in third peak possibly contributing toward exponential rise in daily cases and fatal outcomes. Only 39.2% of these common mutations were seen during second peak, of which only 8.1% were exclusive to second peak. Although 16.2% of these common mutations were part of the mutation repertoire of first peak but surprisingly no common single mutation was exclusive to the first peak (Fig. 9A). This possibly indicates that the novel mutations arose during first peak but declined in frequency across subsequent peaks.

Furthermore, when we looked at the most frequent mutations (S:D614G, NSP3:F106F, NSP12b:P314L and 5'UTR:241), which were present in more than 90% of the sequences across all peaks and almost all lineages (Fig. S5 in the supplemental material). These four mutations appear to dominate the mutation repertoire throughout the world and have been increasing gradually since the start of the pandemic (9). No other mutation except these four was present in even half of the samples during first and second peaks. The mutation D614G in S gene was found in all the lineages in our data except one sample where B.1.393 was detected. The mutation at 14408C>T in RdRp gene (RNA-dependent RNA polymerase) leading to NSP12b:P314L change has been found to be associated with S:D614G mutation and the structural analysis suggested that it would increase protein stability (17). The mutation, 3037C>T in NSP3, a predicted phosphoesterase, papain-like proteinase causing a synonymous change of F106F was found in all the 26 lineages in our data across all peaks. The 4th most common mutation, 241C>T in 5`UTR region is important for the genomic replication process (18, 19) (Fig. 9B).

During third peak in addition to these four, 13 more mutations were present in more than 80% of samples (Fig. 9B). Three more out of these 13 mutations were in 3′ or 5′ UTRs. One of these common mutations during third peak, S:P681R placed at a fur in cleavage position that separates the spike 1 (S1) and S2 subunits is known to increase infectivity of the Delta variant via cell surface entry (20). Similarly, the other most common mutation during third peak *viz.* S:D950N in the S2 region may contribute to the regulation of spike protein (21). Substitution mutation 29742G>T in 3'UTR conserved region (commonly detected during third peak) known as 3′ stem-loop II-like motif and plays a vital role in viral replication and invasion via enhanced stability of 3′ UTR and its interaction with 5′ UTR (22, 23). G210T in 5'UTR present at low frequency of 4–5% during first (in lineages B.1, B.1.36, B.1.468) and during second peak (in lineages B.1 and B.1.617.2). Its frequency jumped to almost 60% during a period before third peak (present combined in lineages AY.12, AY.4, B.1.1.7, B.1.617.1 and B.1.617.2). Then this mutation was detected in at least 80% of samples during third peak and in 100% of samples after third peak was over.

The sole deletion frequently observed was ORF8:D119 leading to deletion of aspartic acid and is known to make ORF8, a fast-evolving protein involved in immune evasion (24). The mutation 26767T>C leading to amino acid change I82T in the M gene was found in primarily in B.1.36 in first peak and in B.1.617.2 during second peak and B.1.617.2 and its sub-lineages AY.12 and AY.4 in third peak. Retention of this mutation across peaks despite lineages switching indicates a significant role in case burden (I82T is a mutation within third transmembrane helical domain, believed to attach to and transport glucose) (25).

We noticed that within the same lineages in two continuing peaks, the mutational load substantially increased. This pattern holds true for multiple lineages with one exception (B.1.617.1). It further strengthens our observation that lineages over time are enriching their mutation repertoire. (Fig. 9C).

Interestingly we also observed that on days when overall cases were not on the rise, the mutation load was still increasing with time, which possibly indicates that the lineages in low-level circulation were silently accruing mutations despite lack of high case burden. For example, the daily mutation load/sample in B.1.36 was 13.2 during first peak but increased to 20.2 during second peak. Similarly, in B.1.617.2 the daily mutation load/sample continuously rose from 24.3 during second peak to 35.9 in third peak and to 40.3 after the third peak had subsided. This shows that case burden and time for which the virus remains in circulation plays an important role toward rise of newer mutations (Fig. 9D).

It was also noted that when gene-wise mutation burden was compared between two dominant lineages, a consistent pattern of over-abundance of mutations in the B.1.617.2 was observed. For E gene no mutation was shared between two lineages. All of the 14 mutations present in M gene was present in B.1.617.2 with only one mutation M:I82T (present across all samples between two lineages and peaks) shared with B.1.36 (Fig. S6 in the supplemental material). As mentioned previously this is the worth highlighting again that this shared mutation is present in high frequency in both the lineages. We also noted that a substantial proportion of S gene mutations (94.6%) in B.1.617.2 were acquired after second peak. As far as clinical characteristics are concerned, greater accumulation of mutations in B.1.167.2 compared with B.1.36 could have contributed toward more severe symptoms and increased fatality as seen during third peak in Delhi.

Hence, we conclude that molecular epidemiology of SARS-CoV-2 needs to be studied continuously to track change in the amino acids. In addition, effect of such mutations in the disease transmission dynamics and pathophysiology must be promptly assessed. Recent news of emergence of a new variant “B.1.1.529- Omicron” which contains heavily mutated S gene (∼30 mutations) is making waves and has spread to approximately 30 countries around the world and counting including India (25). The pandemic is constantly changing, and we believe many novel mutations and lineages potentially more infectious and/or fatal are bound to occur in near future.

### Limitation of the study.

This is a single center study and clinical details fetched from the specimen referral form were limited. Hence genomic studies with larger sample size integrating data detailing patient’s clinical course are warranted.

### Conclusion.

To the best of our knowledge, this is the first study in which the data from three consecutive peaks of COVID-19 pandemic in Delhi, has been analyzed. The findings from our study suggest that lineage tracking and emergence of novel mutation is of paramount importance. Thus, it is strongly believed that we need faster sequencing in a greater number of samples in order to be ahead of the curve in the fight with SARS-CoV-2.

## MATERIALS AND METHODS

The Department of Clinical Virology, at the Institute of Liver and Biliary Sciences, New Delhi (ILBS) is an ICMR (Indian Council of Medical Research) designated COVID-19 diagnostic facility and a satellite site for INSACOG (Indian SARS-CoV-2 Genomics Consortium). Respiratory specimens (combined nasopharyngeal and oropharyngeal swabs in Viral Transport Medium) tested for COVID-19 in the laboratory from 1 June 2020 to 3 August 2021 were included in the present study. Combined nasopharyngeal and oral swabs were collected from suspected cases of SARS-CoV-2 infection in viral transport media tubes (2 mL) and was subjected to COVID-19 RT-PCR assay for diagnosis. For NGS, RNA elutes after extraction from the viral transport media were used. For the purpose of the study, the time period of different peaks in Delhi was defined as follows: first peak: June to July 2020; second peak: November to December 2020; and third peak: April to July 2021.

A total of 1,23,378 samples were tested from 1 June 2020 to 3 August 2021 among which 15,652 (12.6%) were found positive for COVID-19. Among these positives, a subset of 747 (4.7%) samples representing all three peaks of infection having a cycle threshold (Ct) of  ≤ 30 on reverse transcriptase real-time PCR (RT-PCR) were randomly selected from our in-house database and subjected to whole-genome sequencing.

### Clinical analysis.

Complete demographic and clinical details of patients infected with SARS-CoV-2 were recorded from the specimen referral form issued by ICMR. Cases were divided into different age groups: ≤10, 11–25, 26–50, and >50 years.

Outcomes of all the enrolled cases were recorded via telephonic interviews. The cases were classified into various groups as per WHO clinical definition (26).

### SARS-CoV-2 RT-PCR testing.

Total viral RNA was extracted using 300 μL of specimen using Chemagic Viral DNA/RNA kit (PerkinElmer, Waltham, MA, USA) in a Chemagic 360 instrument (PerkinElmer, Waltham, MA, USA) following manufacturer’s instructions. A 10 μL of the extracted viral RNA elute was further subjected to RT-PCR for the detection of SARS-CoV-2 using RealStar SARS-CoV-2 RT-PCR (Altona Diagnostics, Germany) targeting E gene and S gene. All the positive specimens were grouped into three categories based on Ct values obtained: low (Ct ≤20), medium (Ct 21–25) and high (Ct 26–30).

### SARS-CoV-2 whole-genome sequencing.

Sequencing of the viral isolates was done by Illumina COVIDSeq protocol on NextSeq 550 platform as per the manufacturer’s instructions. The quality check of the prepared libraries was performed using DNA high sensitivity assay kit on Bioanalyzer 2100 (Agilent Technologies, United States). The concentration of the libraries was assessed on Qubit (Thermo Fisher Scientific Inc., USA). For amplification and cDNA conversion steps during library preparation, Veriti 96-Well Thermal Cycler (Applied Biosystems).

### Quality control, mapping of sequences, and lineage assignment.

The raw data in the form of binary base call format (.bcl files) was generated from the NextSeq 550 instrument. These raw files were converted, demultiplexed to fastq file using bcl2fastq (Illumina, v2.20) and were aligned against the SARS-CoV-2 reference genome (NC_045512.2). The alignment of unmapped reads to a reference genome and generation of a consensus genome sequence was done within the custom Illumina BaseSpace Sequence Hub. The lineages nomenclature for each sequence was retrieved using Illumina DRAGEN COVID Lineage App (v3.5.3) following the default parameters. The minimum accepted alignment score was set to 12 and results with scores <12 were discarded. The coverage threshold and virus detection threshold were set to 20 and 5 respectively. The variant calling target coverage which specifies the maximum number of reads with a start position overlapping any given position was set at 50.

Among 747 sequenced, 612 (81.9%) samples with genomic coverage > 80% were finally selected for downstream analysis. The median genomic coverage of quality passed samples was 98.8 while the median sequencing depth was 1805 (Supplementary Figs. S7 to S10 in the supplemental material).

### Phylogenetic analysis and mutation profiling.

QC-threshold passed FASTA files containing sequences from 612 SARS-CoV-2 samples and a reference genome of SARS-CoV-2 isolate named Wuhan-Hu-1 (NCBI Reference Sequence: NC_045512.2) was used for generating phylogenetic tree (Table S1 in the supplemental material). To do this, first multiple sequence alignment was done using MAFFT (MAFFT v7.487) with the default options. After performing the alignment, the FASTA file was further refined to perform biologically relevant trimming. Finally, FastME was used for distance-based inference to create an output tree file which was used for visualization of phylogenetic tree. The annotation of nucleotide sequences was done based on NUCMER (Nucleotide Mummer) alignment tool, version 3.1 (a part of the MUMmer package). The mutations are classified according to frequency, the genomic coordinates affected and their subsequent effect on amino acid sequences (Table S2). The retrieved sequences were deposited in the public repository, GISAID (Global Initiative on Sharing All Influenza Data; https://www.gisaid.org) details of which are provided as supplementary data (Table S3). Refer to the supplementary data for extended methodology.

### Data availability.

The raw sequencing data of all the samples have been submitted to the GISAID database and the submission details are given in the Table S3 in the supplemental material. Moreover, the sequences in the FASTA format are also supplied as Table S1.

## References

[1] Zhu N, Zhang D, Wang W, Li X, Yang B, Song J, Zhao X, Huang B, Shi W, Lu R, Niu P, Zhan F, Ma X, Wang D, Xu W, Wu G, Gao GF, Tan W, China Novel Coronavirus Investigating and Research Team. 2020. A novel coronavirus from patients with pneumonia in China, 2019. N Engl J Med 382:727–733. doi:10.1056/NEJMoa2001017. 31978945 PMC7092803

[2] Cucinotta D, Vanelli M. 2020. WHO Declares COVID-19 a Pandemic. Acta Biomed 91:157–160. 32191675 10.23750/abm.v91i1.9397 PMC7569573

[3] Andrews MA, Areekal B, Rajesh KR, Krishnan J, Suryakala R, Krishnan B, Muraly CP, Santhosh PV. 2020. First confirmed case of COVID-19 infection in India: A case report. Indian J Med Res 151:490–492. doi:10.4103/ijmr.IJMR_2131_20. 32611918 PMC7530459

[4] WHO. 2021. India: WHO Coronavirus Disease (COVID-19) Dashboard With Vaccination Data [Internet]. https://covid19.who.int.

[5] Dhar MS, Marwal R, Vs R, Ponnusamy K, Jolly B, Bhoyar RC, Sardana V, Naushin S, Rophina M, Mellan TA, Mishra S, Whittaker C, Fatihi S, Datta M, Singh P, Sharma U, Ujjainiya R, Bhatheja N, Divakar MK, Singh MK, Cherian SS. 2021. Genomic characterization and epidemiology of an emerging SARS-CoV-2 variant in Delhi, India. Science 374:995–999. doi:10.1126/science.abj9932. 34648303 PMC7612010

[6] Li X, Zai J, Zhao Q, Nie Q, Li Y, Foley BT, Chaillon A. 2020. Evolutionary history, potential intermediate animal host, and cross-species analyses of SARS-CoV-2. J Med Virol 92:602–611. doi:10.1002/jmv.25731. 32104911 PMC7228310

[7] Yadav PD, Nyayanit DA, Majumdar T, Patil S, Kaur H, Gupta N, Shete AM, Pandit P, Kumar A, Aggarwal N, Narayan J, Vijay N, Kalawat U, Sugunan AP, Munivenkatappa A, Sharma T, Devi S, Majumdar T, Jaryal S, Bakshi R, Joshi Y, Sahay R, Shastri J, Singh M, Kumar M, Rawat V, Dutta S, Yadav S, Krishnasamy K, Raut S, Biswas D, Borkakoty B, Verma S, Rani S, Deval H, Patel D, Turuk J, Malhotra B, Fomda B, Nag V, Jain A, Bhargava A, Potdar V, Cherian S, Abraham P, Gopal A, Panda S, Bhargava B. 2021. An epidemiological analysis of SARS-CoV-2 genomic sequences from different regions of India. Viruses 13:925. doi:10.3390/v13050925. 34067745 PMC8156686

[8] Pattabiraman C, Prasad P, George AK, Sreenivas D, Rasheed R. 2022. Importation, circulation, and emergence of variants of SARS-CoV-2 in the South Indian State of Karnataka. Wellcome Open Res 6:110. doi:10.12688/wellcomeopenres.16768.2. 35243004 PMC8857524

[9] Urhan A, Abeel T. 2021. Emergence of novel SARS-CoV-2 variants in the Netherlands. Sci Rep 11:6625. doi:10.1038/s41598-021-85363-7. 33758205 PMC7988010

[10] Jain VK, Iyengar KP, Vaishya R. 2021. Differences between first wave and second wave of COVID-19 in India. Diabetes & Metabolic Syndrome. 10.1016/j.dsx.2021.05.009 PMC8106236 33992554

[11] Budhiraja S, Indrayan A, Aggarwal M, Jha V, Jain D, Tarai B, Das P, Aggarwal B, Mishra RS, Bali S, Mahajan M. 2021. Differentials in the characteristics of COVID-19 cases in Wave-1 and Wave-2 admitted to a network of hospitals in North India. medRxiv.

[12] Sarkar A, Chakrabarti AK, Dutta S. 2021. Covid-19 infection in India: A comparative analysis of the second wave with the first wave. Pathogens 10:1222. doi:10.3390/pathogens10091222. 34578254 PMC8469101

[13] Callaway E. 2020. The coronavirus is mutating—does it matter? Nature NLM 585:174–177. doi:10.1038/d41586-020-02544-6. 32901123

[14] Vilar S, Isom DG. 2021. One year of SARS-CoV-2: How much has the virus changed? Biology (Basel) 10:91. doi:10.3390/biology10020091. 33530355 PMC7911924

[15] Baj A, Novazzi F, Ferrante FD, Genoni A, Tettamanzi E, Catanoso G, Gasperina DD, Dentali F, Focosi D, Maggi F. 2021. Spike protein evolution in the SARS-CoV-2 Delta variant of concern: a case series from Northern Lombardy. Emerg Microbes Infect 10:2010–2015. doi:10.1080/22221751.2021.1994356. 34651569 PMC8567936

[16] Volz E, Mishra S, Chand M, Barrett JC, Johnson R, Geidelberg L, Hinsley RW, Laydon JD, Dabrera G, Tool OÁ, Amato R, Ragonnet-Cronin M, Harrison I, Jackson B, Ariani VC, Boyd O, Loman NG, McCrone JT, Gonçalves S, Jorgensen D, Myers J, Hill V, Jackson D, Gaythorpe K, Groves N, Sillitoe J, Kwiatkowski PD, Flaxman S, Ratmann O, Bhatt S, Hopkins S, Gandy A, Rambaut A, Ferguson MN. 2021. Transmission of SARS-CoV-2 lineage B.1.1.7 in England: insights from linking epidemiological and genetic data [Internet]. https://www.medrxiv.org/content/10.1101/2020.12.30.20249034v2.

[17] Haddad D, John SE, Mohammad A, Hammad MM, Hebbar P, Channanath A, Nizam R, Al-Qabandi S, Al Madhoun A, Alshukry A, Ali H, Thanaraj TA, Al-Mulla F. 2021. SARS-CoV-2: Possible recombination and emergence of potentially more virulent strains. PLoS One 16:e0251368. doi:10.1371/journal.pone.0251368. 34033650 PMC8148317

[18] Li T, Zhang Y, Fu L, Yu C, Li X, Li Y, Zhang X, Rong Z, Wang Y, Ning H, Liang R, Chen W, Babiuk LA, Chang Z. 2005. siRNA targeting the leader sequence of SARS-CoV inhibits virus replication. Gene Ther 12:751–761. doi:10.1038/sj.gt.3302479. 15772689 PMC7091583

[19] Rangan R, Zheludev IN, Hagey RJ, Pham EA, Wayment-Steele HK, Glenn JS, Das R. 2020. RNA genome conservation and secondary structure in SARS-CoV-2 and SARS-related viruses: a first look. RNA 26:937–959. doi:10.1261/rna.076141.120. 32398273 PMC7373990

[20] Liu Y, Liu J, Johnson BA, Xia H, Ku Z, Schindewolf C, Widen SG, An Z, Weaver SC, Menachery VD, Xie X, Shi P-Y. 2021. Delta spike P681R mutation enhances SARS-CoV-2 fitness over alpha variant. bioRxiv. 10.1016/j.celrep.2022.110829 PMC9050581 35550680

[21] Planas D, Veyer D, Baidaliuk A, Staropoli I, Guivel-Benhassine F, Rajah MM, Planchais C, Porrot F, Robillard N, Puech J, Prot M, Gallais F, Gantner P, Velay A, Le Guen J, Kassis-Chikhani N, Edriss D, Belec L, Seve A, Courtellemont L, Péré H, Hocqueloux L, Fafi-Kremer S, Prazuck T, Mouquet H, Bruel T, Simon-Lorière E, Rey FA, Schwartz O. 2021. Reduced sensitivity of SARS-CoV-2 variant Delta to antibody neutralization. Nature 596:276–280. doi:10.1038/s41586-021-03777-9. 34237773

[22] Li L, Kang H, Liu P, Makkinje N, Williamson ST, Leibowitz JL, Giedroc DP. 2008. Structural lability in stem–loop 1 drives a 5′ UTR–3′ UTR interaction in coronavirus replication. J Mol Biol 377:790–803. doi:10.1016/j.jmb.2008.01.068. 18289557 PMC2652258

[23] Yang D, Leibowitz JL. 2015. The structure and functions of coronavirus genomic 3′ and 5′ ends. Virus Res 206:120–133. doi:10.1016/j.virusres.2015.02.025. 25736566 PMC4476908

[24] Flower TG, Buffalo CZ, Hooy RM, Allaire M, Ren X, Hurley JH. 2021. Structure of SARS-CoV-2 ORF8, a rapidly evolving immune evasion protein. Proc Natl Acad Sci USA 118:e2021785118. doi:10.1073/pnas.2021785118. 33361333 PMC7812859

[25] Rao S, Singh M. 2021. The newly detected B. 1.1. 529 (Omicron) variant of SARS-CoV-2 with multiple mutations: Implications for transmission, diagnostics, therapeutics, and immune evasion. Dhrp 1:7–10. doi:10.47488/dhrp.v1iS5.35.

[26] WHO. 2021. WHO COVID-19 Case definition [Internet]. https://www.who.int/publications/i/item/WHO-2019-nCoV-Surveillance_Case_Definition-2020.2.

