# Supplementary material for: SARS-CoV-2 Lineage Tracking, and Evolving Trends Seen during Three Consecutive Peaks of Infection in Delhi, India: a Clinico-Genomic Study
Source: Microbiol Spectr. 2022 Mar 21;10(2):e02729-21. doi: 10.1128/spectrum.02729-21 (PMC9045110; doi:10.1128/spectrum.02729-21)

**Supplementary Methods.**

## 1. Clinical classification of samples into various groups as per WHO definition.

**1. Asymptomatic group:** RT-PCR positive patient without any clinical symptoms of upper or lower respiratory tract illness or impaired daily activities.

**2. Symptomatic group:** RT-PCR positive patient presenting with clinical symptoms of either upper or lower respiratory tract infection like fever, cough, sore throat, body ache or headache, breathlessness, diarrhoea etc. and with impaired daily activities.

Symptomatic patients were further sub-divided based on the severity of illness

A. **Influenza-Like Illness (ILI)**– is defined as acute respiratory infection with a measured fever of ≥38°C and cough, with onset within the past ten days.

B. **Severe Acute Respiratory Illness (SARI)**– is defined as – history of fever or measured fever of ≥38°C and cough, with acute onset within the past ten days and require hospitalization.

## 2. Statistical analysis

All the statistical analysis and plotting were done in R environment, version 4.1.1 (R Core Team (2020)[10] and using custom-made scripts. The statistical testing for checking data normality was performed by the Shapiro-Wilk test. For categorical variables Pearson's $\chi 2$ test with Yates’ correction and for comparison across two/more than two groups in case of continuous data, non-parametric Wilcoxon rank sum test/Kruskal-Wallis rank-sum test was utilized respectively). Tukey HSD post hoc multiple comparison tests were used after ANOVA/ Kruskal-Wallis test yielded significant p values to see which among the three or more groups are significantly different.

**Supplementary Figure S1. Distribution and comparison of different age- groups across three peaks.**

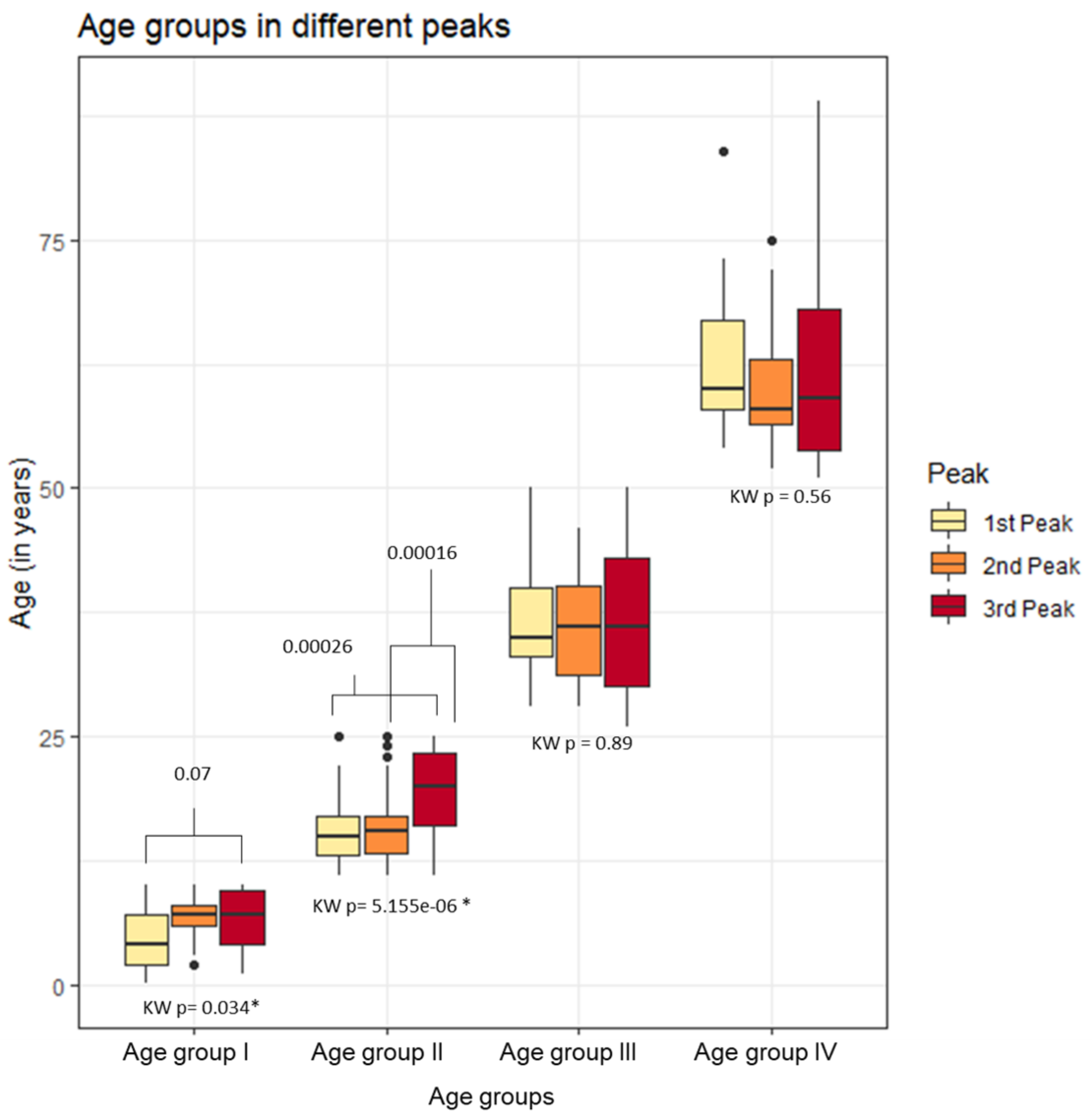

**Supplementary Figure S2. Distribution and comparison of Ct values across three peaks.**

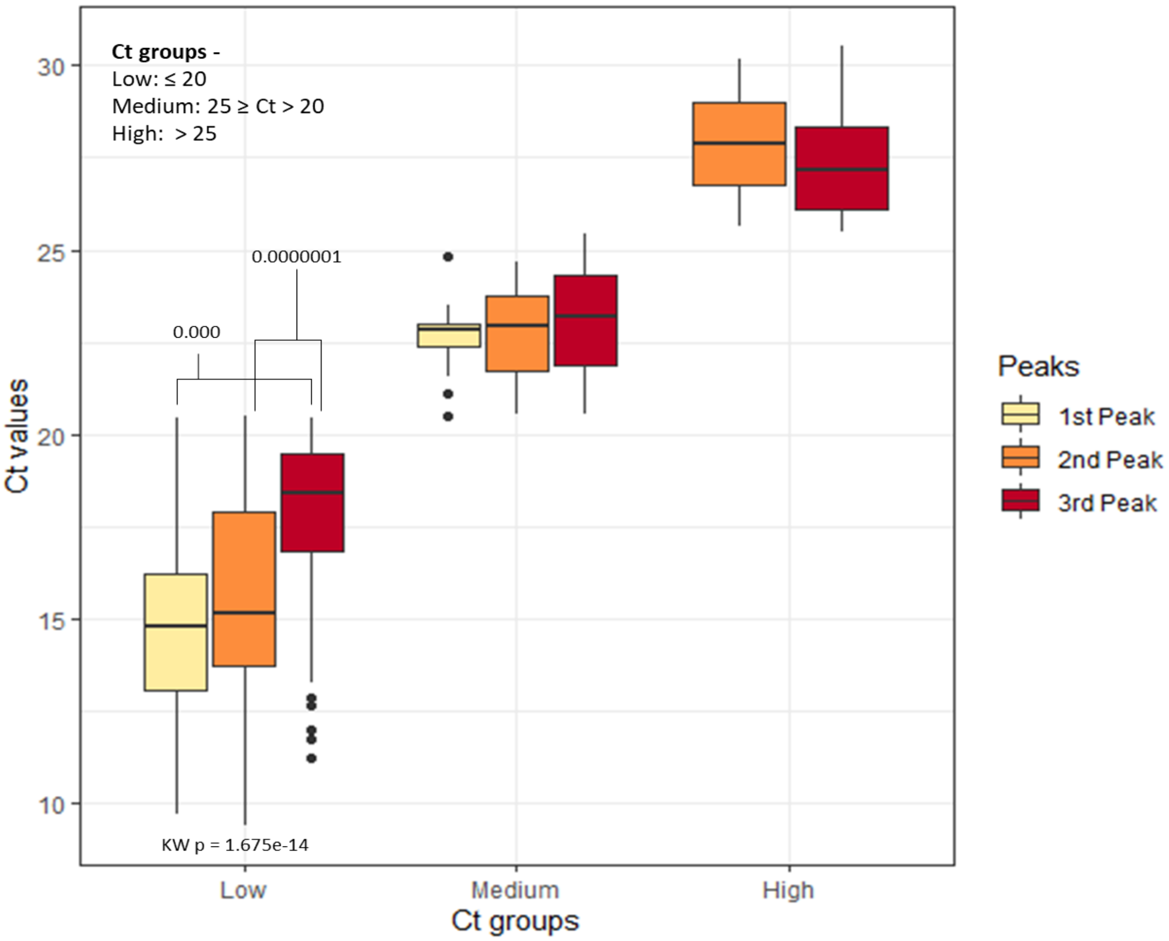

**Supplementary Figure S3**. **Nucleotide variations reported from 612 samples.** The red cut-off line represents the frequency of 0.1 (10%).

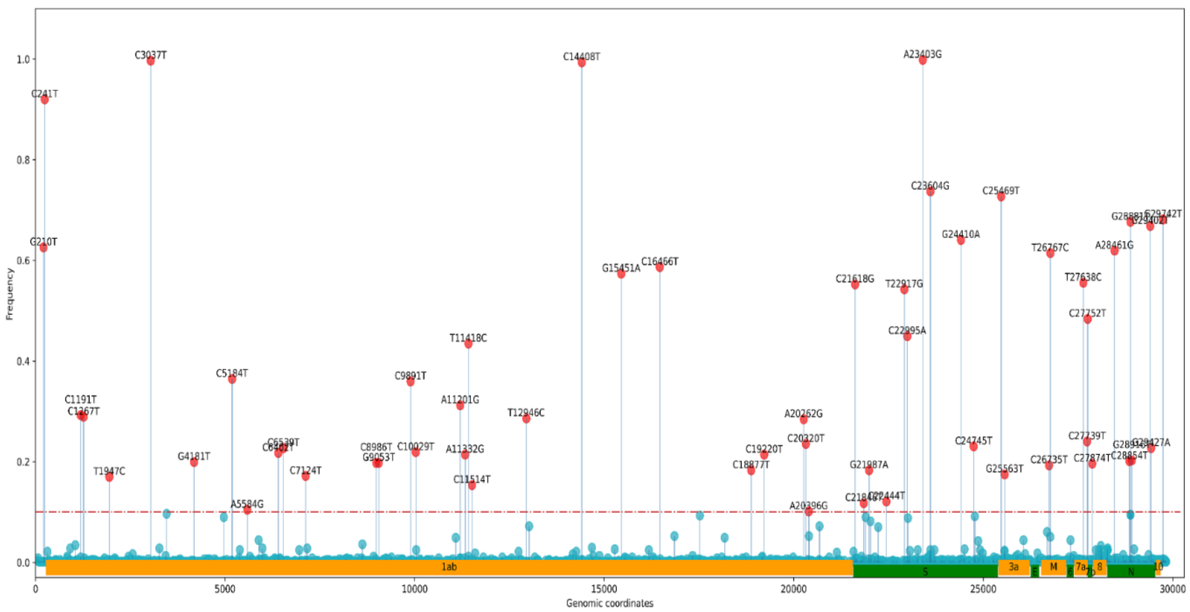


**Supplementary Figure S4**. **Synonymous and Non-synonymous SNPs in different SARS-Cov-2 genes** (UTR and indel events are not displayed here)

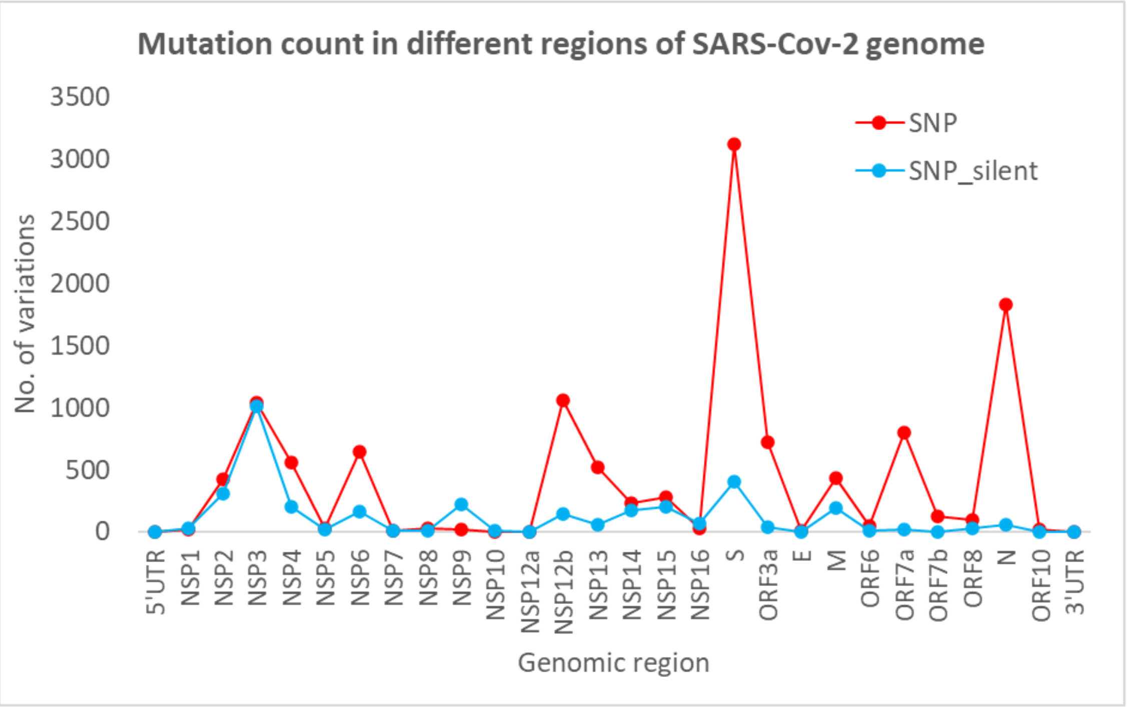

**Supplementary Figure S5.** The timeline profile of four of the most common mutations: S:D614G, NSP3:F106F, NSP12b:P314L and 5'UTR:241 in different lineages. Broadly the four mutations followed a similar pattern and dominated the pandemic time period

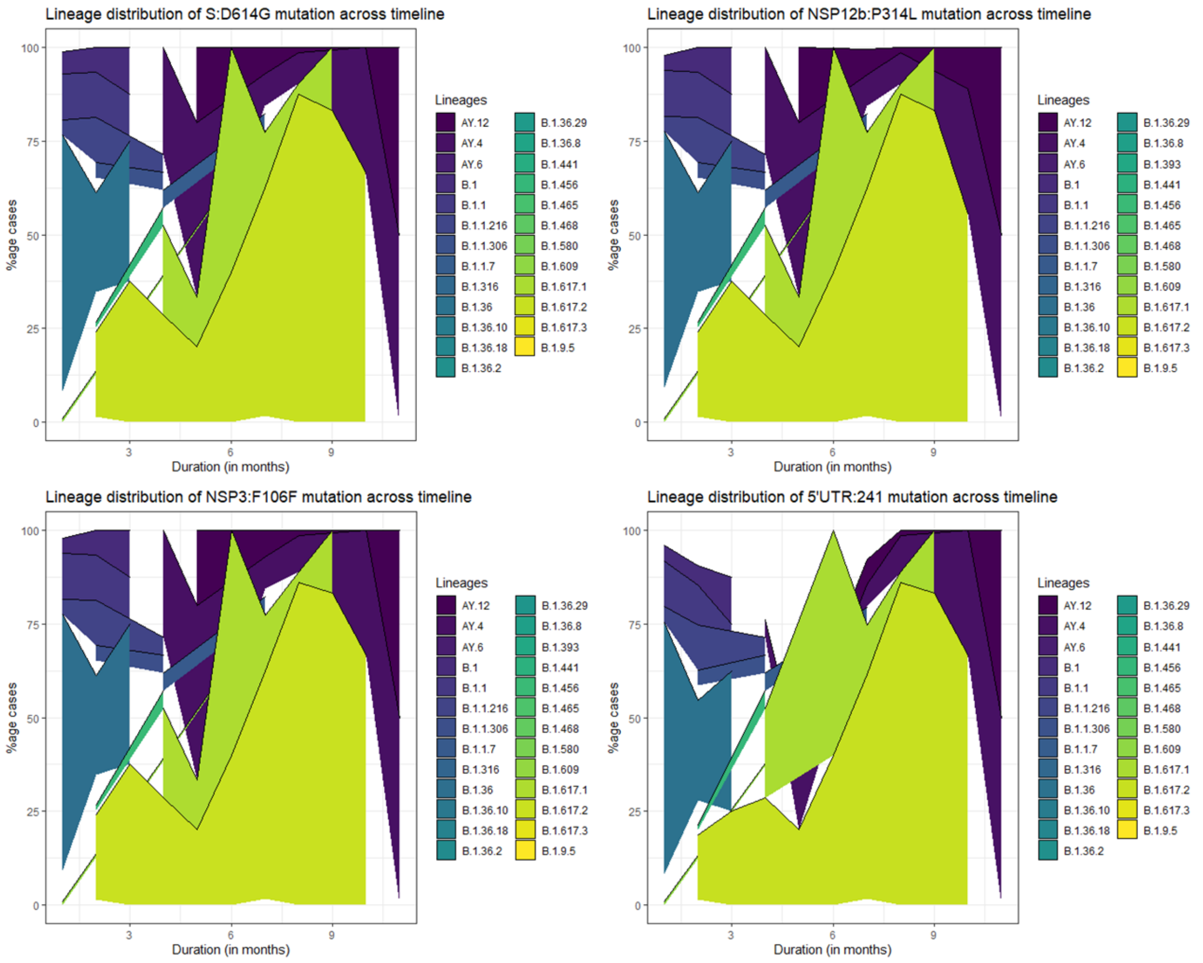

**Supplementary Figure S6**. Comparison between mutation profile of B.1.36 and B.1.617.2 lineages in different genes

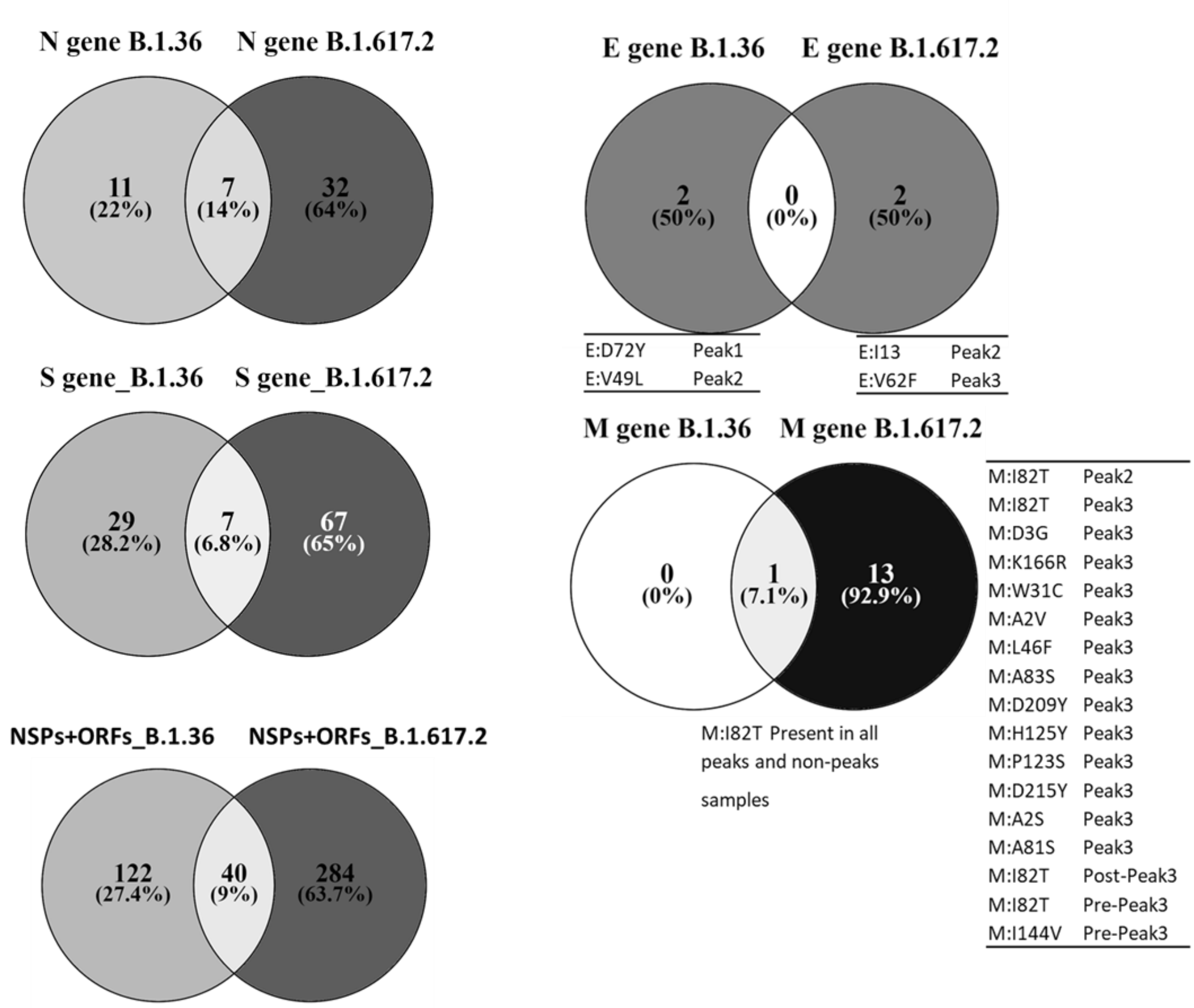

**Supplementary Figure S7**. Sequence Quality Histograms. The mean quality value across each base position in the read for all sequences.

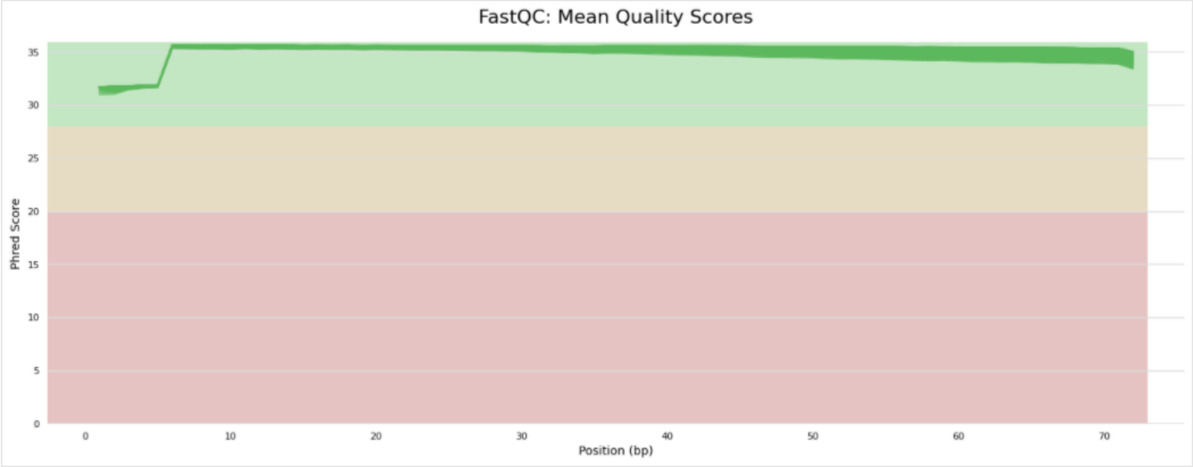


**Supplementary Figure S8**. Per Sequence Quality Scores. The number of reads with average quality scores.

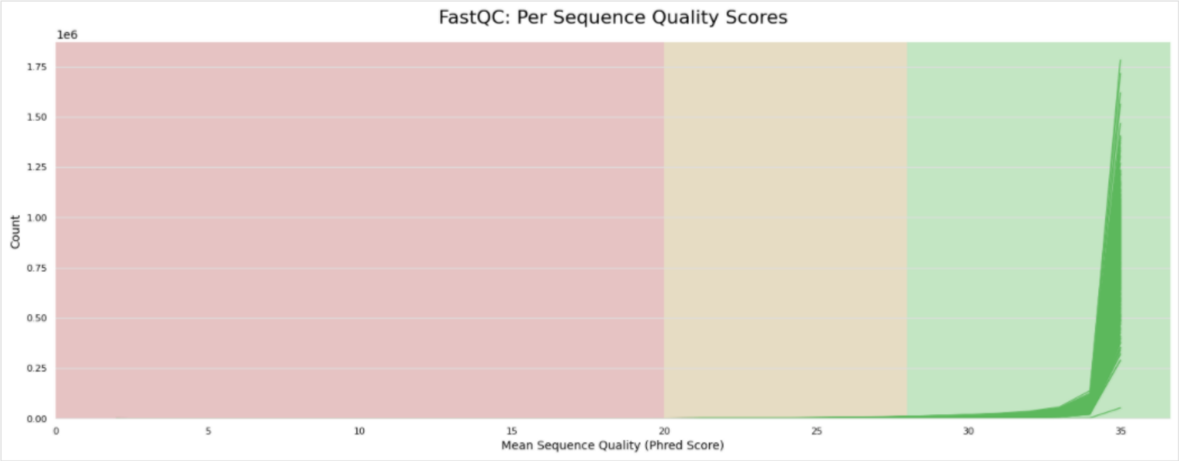

**Supplementary Figure S9**. Per Base N Content. The percentage of base calls at each position for which an N was called.

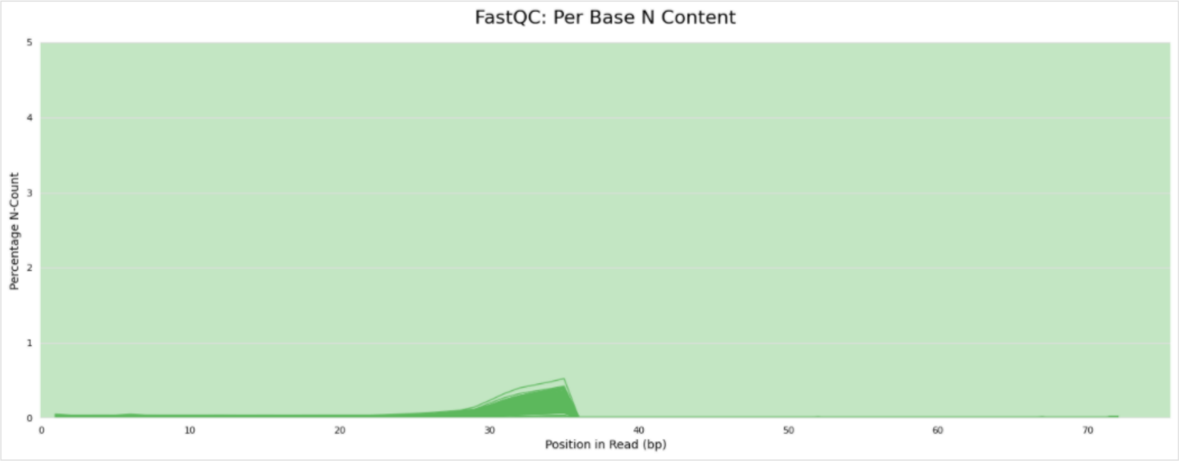


**Supplementary Figure S10.** Sequence Length Distribution. The distribution of fragment sizes (read lengths).

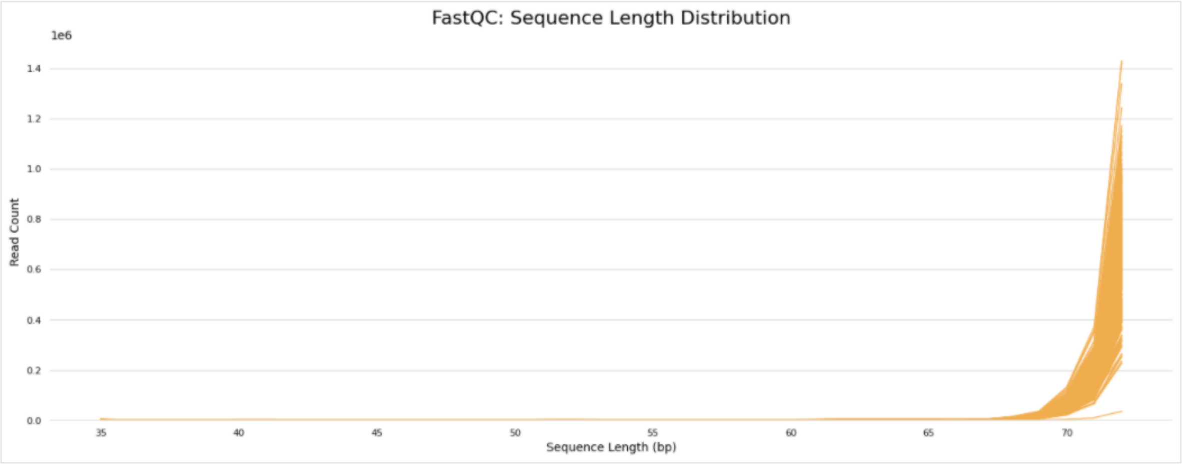

**Supplementary Figure S11.** The schematic for the work flow of the study

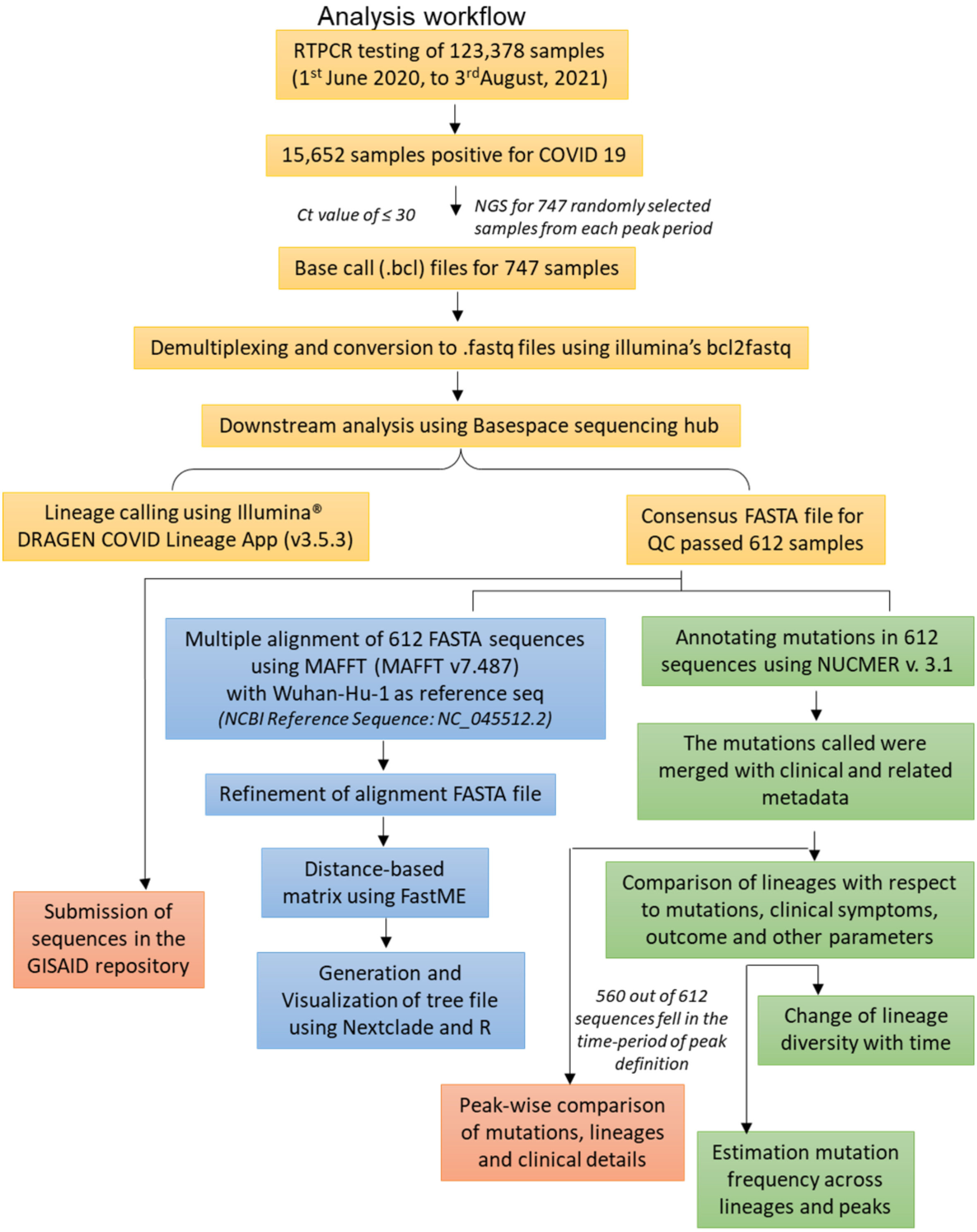

Supplement: SUPPLEMENTAL FILE 1 — Supplemental material. Download SPECTRUM02729-21_Supp_1_seq12.pdf, PDF file, 1.4 MB [file spectrum02729-21_supp_1_seq12.pdf]
